# Effectiveness of the application of an educational program based on the Health Belief Model (HBM) in Adopting Preventive Behaviors from Self-Medication among Women in Iran. A Randomized Controlled Trial*

**DOI:** 10.17533/udea.iee.v40n3e11

**Published:** 2023-02-13

**Authors:** Ehsan Movahed, Monireh Rezaee Moradali, Mohammad Saeed Jadgal, Morad Ali Zareipour, Mina Tasouji Azari

**Affiliations:** 1 Department of Public Health, School of Public Health, Jiroft University of Medical Sciences, Jiroft, Iran . Email: ehsan.movahed@yahoo.com University of Medical Sciences Department of Public Health School of Public Health Jiroft University of Medical Sciences Jiroft Iran ehsan.movahed@yahoo.com; 2 Urmia Branch, Islamic Azad University, Urmia, Iran. Email:monir.rezaee@yahoo.co.uk Islamic Azad University Islamic Azad University Urmia Iran monir.rezaee@yahoo.co.uk; 3 Tropical and communicable diseases research center, Iranshahr University of Medical Science and Health Services, Iranshahr, Iran. Department of Public Health, School of Nursing, Iranshahr University of Medical Sciences, Chabahar, Iran. Email: jadgal15@gmail.com. Iran University of Medical Sciences Iranshahr University of Medical Science and Health Services Iranshahr Iran jadgal15@gmail.com; 4 Department of Public Health, School of Health, Khoy University of Medical Sciences, Khoy, Iran. Email: zareipoor_m@khoyums.ac.ir. Corresponding author Iran University of Medical Sciences Department of Public Health School of Health Khoy University of Medical Sciences Khoy Iran zareipoor_m@khoyums.ac.ir; 5 Department of English Language, Islamic Azad University, Tabriz, Iran. Email: minatasoujii@yahoo.com Islamic Azad University Department of English Language Islamic Azad University Tabriz Iran minatasoujii@yahoo.com

**Keywords:** health belief model, behavior, self medication, women., modelo de creencias sobre la salud, conducta, automedicación, mujeres., modelo de crenças de saúde, comportamento, automedicação, mulheres.

## Abstract

**Objective.:**

To evaluate the effectiveness of the application of an educational program based on the Health Belief Model (HBM) in Adopting Preventive Behaviors from Self-Medication among Women in Iran.

**Methods.:**

Interventional study with pre and post phases. 200 women referring to the health centers of Urmia were selected by simple random sampling, divided into two groups of treatment and control. Data collection instruments were researcher-devised questionnaire including the questionnaire of Knowledge of Self-medication, the Questionnaire of Preventive Behaviors from Self-medication, and the questionnaire of Health Belief Model. The questionnaires were assessed for expert validity and then, were checked for reliability. The educational intervention was conducted for the treatment group during four weeks four 45-minute sessions.

**Results.:**

The average scores of knowledge, perceived susceptibility, perceived severity, perceived benefits, perceived barriers, cues to action, self-efficiency, and post-intervention performance in have increased in treatment group, comparing to the control group, All findings were statistically significant (*p*<0.05). Furthermore, social media, doctors, and disbelief in self-medication were more effective in increasing awareness and encouraging to have proper medication, also, the highest self-medication was in taking pain-relievers, cold tablets and antibiotics, which showed significant decrease in treatment group after the intervention.

**Conclusion.:**

The educational program based on Health Belief Model was effective in reducing the self-medication among the studied women. Furthermore, it is recommended to use social media and doctors to improve the awareness and motivation among people. Thus, applying the educational programs and plans according to the Health Belief Model can be influential in reducing the self-medication.

## Resumen

**Objetivo.:**

Evaluar la eficacia de la aplicación de un programa educativo basado en el Modelo de Creencias sobre la Salud (MCS) en la adopción de conductas preventivas de la automedicación entre las mujeres de Irán.

**Métodos.:**

Estudio de intervención con evaluación pre y post. Se seleccionaron 200 mujeres que acudieron a los centros de salud de Urmia, a quienes se asignaron a los dos grupos de estudio (tratamiento y control) mediante un muestreo aleatorio simple. Para la recolección de la información se utilizaron los cuestionarios sobre: Conocimientos acerca de la automedicación, conductas preventivas de la automedicación y el modelo de creencias sobre la salud. Se evaluó la validez de los cuestionarios por parte de los expertos y luego se comprobó su confiabilidad. La intervención educativa se llevó a cabo para el grupo de tratamiento durante cuatro semanas con 1 sesión semanal de 45 minutos de duración.

**Resultados.:**

Las puntuaciones medias de los conocimientos, la susceptibilidad percibida, la gravedad percibida, los beneficios percibidos, las barreras percibidas, las señales para la acción, la autoeficacia y el rendimiento posterior a la intervención aumentaron en el grupo de tratamiento en comparación con el grupo de control, y todos los resultados fueron estadísticamente significativos (*p*<0.05). Además, los medios de comunicación social fueron eficaces para aumentar la concienciación y animar a tener una medicación adecuada. La mayor automedicación fue en la toma de analgésicos, pastillas para el resfriado y antibióticos, que mostró una disminución significativa en el grupo de tratamiento después de la intervención.

**Conclusión.:**

El programa educativo basado en el Modelo de Creencias de Salud fue eficaz para reducir la automedicación entre las mujeres estudiadas. Además, se recomienda utilizar los medios de comunicación social para mejorar la concienciación y la motivación de las personas.

## Resumo

**Objetivo.:**

Avaliar a eficácia da aplicação de um programa educativo baseado no Modelo de Crenças em Saúde (HCM) na adoção de comportamentos preventivos de automedicação entre mulheres no Irã.

**Métodos.:**

Estudo de intervenção com pré e pós avaliação. Duzentas mulheres que frequentavam os centros de saúde de Urmia foram selecionadas e alocadas nos dois grupos de estudo (tratamento e controle) por meio de amostragem aleatória simples. Para a coleta de informações, foram utilizados os questionários sobre: Conhecimento sobre automedicação, comportamentos preventivos de automedicação e o modelo de crenças sobre saúde. A validade dos questionários foi avaliada pelos especialistas e, em seguida, verificada sua confiabilidade. A intervenção educativa foi realizada para o grupo de tratamento durante quatro semanas com 1 sessão semanal com duração de 45 minutos.

**Resultados.:**

Os escores médios de conhecimento, suscetibilidade percebida, gravidade percebida, benefícios percebidos, barreiras percebidas, pistas para ação, autoeficácia e desempenho pós-intervenção aumentaram no grupo de tratamento em comparação com o grupo de intervenção. controle, e todos os resultados foram estatisticamente significativos (*p*<0.05). Além disso, as mídias sociais foram eficazes na conscientização e no incentivo à medicação adequada. A maior automedicação foi em uso de analgésicos, antissépticos e antibióticos, que apresentou diminuição significativa no grupo de tratamento após a intervenção.

**Conclusão.:**

O programa educativo baseado no Modelo de Crenças em Saúde foi eficaz na redução da automedicação entre as mulheres estudadas. Além disso, recomenda-se o uso das mídias sociais para melhorar a conscientização e a motivação das pessoas.

## Introduction

Healthy human is regarded as the basis of sustainable development, in which the role of medication has proven to be primary, effective and determinant.(1) As the most common form of self-care, self-medication is defined as taking medication without a doctor's prescription, using medications prescribed for other family members, refusing to take the original prescribed medication, and overusing over-the-counter under-medication medications.(2,3) The consequences of Self-medication may include complexities like disturbance in drug market, very high expenses of medicine budgeting of the government, the delay in treatment of a sever disease, development of drug resistance, no optimal treatment, poisoning, unwanted consequences and eventually may leading to death.(1) These days, according to Panchal et al., arbitrary drug use and self-medication in general, is considered as one of the biggest social, economic and health problems of different societies including Iran.(4) In the World Health Day Slogan in 2011 was declared the resistance to anti-bacterial drugs as a global threat. During a study on Italian families, 69% had arbitrary drug use at least once (6), and in the study done by Pavan, 5% of people had experienced arbitrary drug use.(5) The amount and range of self-medication is different in different cities of the country, so that it is reported as 94% in Ahvaz,(7) 63% in Tabriz,(8) 86% in Isfahan,(9) 54% in Arak,(10) and 83% in Yazd.(11)

In the meantime, paying attention to the population of women has great importance, as their experiencing some critical periods like pregnancy and breast feeding, more being in contact with family members, and being regarded as role models and examples for other family members. Different studies show that women show particular tendency to have self-medication, so arbitrarily and frequently use drugs to cure problems like dysmenorrhea, to eliminate symptoms of menopause, menstrual disorders, mood disorders, as well as the problems occur in pregnancy and breast feeding, all can bring about self-medication among women.(12) 

The Health Belief Model (HBM) was one of the first behavior change models to explain health decision-making and the consequences of behavior, which was proposed by social psychologists in the 50s to explain people's desire to adopt preventive behaviors. After making corrections and adding new structures to this model, it was used to identify people's behavior in the field of disease prevention, screening and control.(13) 

The constructs of the HBM consist of perceived susceptibility, perceived severity, perceived benefits, perceived barriers, self-efficacy, and cues to action. According to this model, a person's decision to adopt a behavior depends on the person's perception and how much they consider themselves to be at risk and prone to disease (perceived susceptibility); then understand the depth of clinical, medical, and social consequences (perceived severity); With the cues and stimuli they receives (cues to action), they believe in the benefits and applicability of preventive behavior (perceived benefits) and finds the factors that prevent the behavior to be low-cost (perceived barriers) and sees himself as capable of performing the preventive behavior (perceived self-efficacy) to finally choose the correct behavior.(13) In this vein, the findings of the studies on applying the HBM model aiming at increasing the physical activity, adopting preventive behaviors from Alzheimer, prove the health promoting self-care behaviors manifesting their efficiency.(14,15) Regarding the ever-increasing widespread occurrence of self-medication in communities, and the direct role of the individual in drug selection and use in order to have a longer life with a fairly healthy and active living, it seems urgent to determine the effective factors on. Therefore, the purpose of the present study is to evaluate the effectiveness of the application of an educational program based on the Health Belief Model (HBM) in Adopting Preventive Behaviors from Self-Medication among Women in Iran.

## Methods

The present study is an interventional and semi-experimental one, conducted among women in Urmia in 1398 (2018) with the purpose of applying Health Belief Model (HBM) in adopting preventive behaviors from self-medication of women. Having regarded the previously conducted studies, the prevalence of self-medication was estimated to be 36%,(16) which has been calculated with α=5%, 95% confidence level, and d=6%, on the sample size of 200 people, of which taking 100 for each groups of control and intervention. In the formula, p=36%, q=64%, d=6% and z=1.96. 




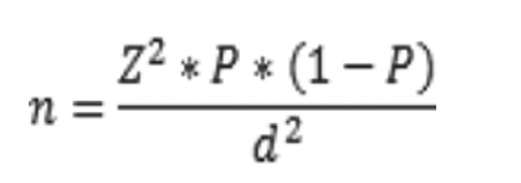




The sampling was the stratified sampling way performed by referring to the health centers of the city. The samples were selected randomly from women referred to 10 defined health centers, then every one of the centers were placed in the control and intervention groups (5 control centers and 5 intervention centers). The sampling in the clinics was done randomly according to the household codes. The way of sampling in each clinic was simple and random as well, according to the code of the household available at the centers, as these samples were invited to a meeting on a definite day to the health center with the objective of getting acquainted, getting informed of the purpose of the study, as well as receiving the written informed agreement and consent of participation in the study. Inclusion criteria included consent to participate in the study, having a health record at the health center, and not having a specific disease. Exclusion criteria were women who were unable to cooperate. The whole population of people taking part in the present study accounted for 200 people, of whom 18 people didn’t fill in the questionnaire, thus more women were added to the study to have 200 accomplished questionnaires at the end.

**Figure:**
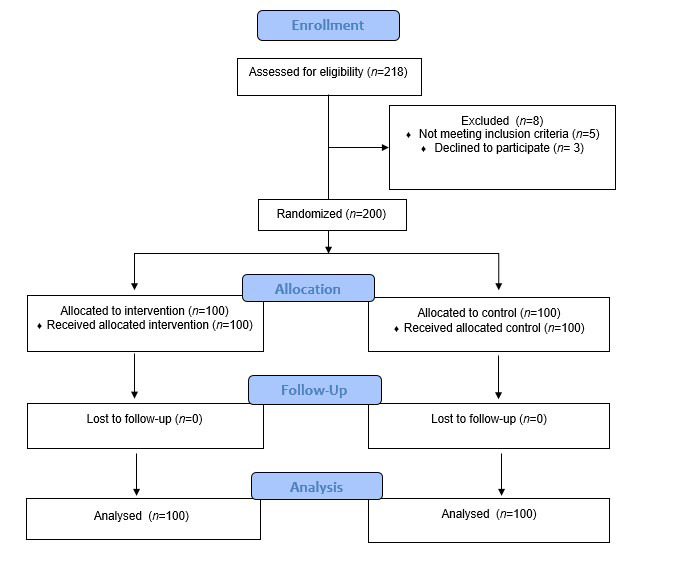

The data collection instruments include researcher-devised questionnaire including the questionnaire of knowledge of self-medication, the questionnaire of preventive behaviors from self-medication, and the questionnaire of Health Belief Model. The items relevant to each indicated consequences were selected according to the literature review, and to meet the validity and reliability of the questionnaire, the approaches of content validity and Alpha-Cronbach Test were implemented respectively. To measure the validity of the questionnaire, it was sent to 10 experts in the health education and gynecologists, then the necessary modifications were applied on the basis of their comments. The validity was measured to be above 80%. To assess the reliability of the questionnaire, it was responded by 30 women, then by using Cronbach Alpha Test, the reliability coefficient of the questions of Knowledge was set as 0.77%, Perceived Sensitivity 0.75%, Perceived Severity 0.82%, Perceived Barriers 0.86%, Cues to Action 0.81%, Perceived Benefits 0.84%, and Self-efficiency as 0.76%.

Assessing Knowledge was done in the form of 12 questions with Yes/No/Don’t Know options, as the respond of ‘Yes’ receiving 2 points, ‘Don’t Know’ getting 1 point, and ‘No’ having zero. The scores of Knowledge Questionnaire varied from zero to 24. Next, the questions and scores of the Health Belief Model include all questions on the basis of Likert scale with three options of “Agree, No Idea, and Disagree”. Because of the more complex understanding of the area being assessed, the ‘Perceived Sensitivity’ had 5 questions with maximum and minimum of 5-15 points, ‘Perceived Severity’ had 6 questions with 6-18 points, ‘Perceived Barriers” had 5 questions 5-15 points, ‘Perceived Benefits’ having 7 questions with 7-21 points, ‘Self-efficiency’ having 10 questions 10-30 points, ‘Cues for Action’ having 7 questions with 7-21 points. To measure the preventive behaviors from self-medication, 10 questions were applying as well, with the options of ‘Always Yes’, ‘Sometimes Yes’, and ‘No’, as the response of ‘Always Yes’ receiving 2 points, ‘Sometimes Yes’ with 1 point and ‘No’ having zero point. The scores of the questionnaire of the preventive behaviors from self-medication varied from zero to 20.

The expected intervention was performed according to the Health Belief Model for the intervention group, including 4 educational sessions for 45 minutes. As the number of the participants in the control group was 100 people, the educational classes were divided into 20 people classes, having 4 educational sessions on the basis of Health Belief Model.

The implemented educational methods were lecturing, asking and answering questions, and group discussion, while in order to assist women’s better understanding, to prevent misunderstanding, and to involve their visual learning as its critical importance, other educational equipment and materials like posters, educational pamphlets, booklets and whiteboard were implemented as well. The educational content and materials were prepared on the basis of educational goals, the needs-analysis conducted earlier, and regarding the valid books and pamphlets available, as well as the pharmaceutical monthly magazine titled “Razi”, and counselling the pharmaceutical specialists. The first session consisted the history of self-medication, and improving the knowledge of people on self-medication, the next sessions were on the basis of Health Belief Model, including education to raise the severity, self-efficiency, benefits and barriers on the issue, together with external and internal cues to action regarding self-medication or arbitrary use of drugs. It is worthy of noticing that the presented materials were written in a pamphlet and educational manuals submitted to the participants, then were reviewed and summarized the following session briefly. 

**Table1. Educational:** content on the basis of Health Belief Model regarding self-medication

Sessions(each 45-min)	The title of educational subject	Educational content	Sessions(each 45-min)
Session 1	Knowledge	Knowing about self-medication, self-medication among women, prevalence of self-medication among women, the causes and factors of self-medication, reasons and factors leading to self-medication, preventing ways for self-medication	Session 1
Session2	Perceived susceptibility	Stating the prevalence frequency of self-medication among women, created physiologic changes in women, increasing the possibility of drug-resistance, feeling the menace of being exposure to disease, feeling the risk of self-medication, feeling the need to modify medication	Session2
	Perceived severity	Stating the consequences of self-medication in the physical, psychological, social, and economic aspects, not being able to do the assigned responsibilities after self-medication, cost of medication and hospitalization afterwards, the way of occurrence long-term and short-term complications, highlighting the severity of the consequences of self-medication	
Session 3	Perceived benefits	Identifying the benefits of not doing self-medication, expressing the positive effects of preventing self-medication, on each mentioned consequence of self-medication (reducing the medication expenses, preventing the harm and damage, preventing having some disabilities, not being dependent on others, ability to participate in social events, preventing from staying home, the ability to do every day and recreational activities	Session 3
	Perceived barriers	Trying to persuade the reduction in perceived medication expenses and not self-medication, lectures, discussion and exchange comments on perceived expenses of self-medication with the group members, providing some educational solutions to minimize the perceived expenses, counceling and discussing with the heads of the families on the perceived expenses, persuading them to have enough time to visit the doctor and medication according to the doctor’s prescription	
Session 4	Perceived self-efficacy	Defining the meaning of the self-efficiency and its significance in preventing self-medication, verbal encouragement to promote the feeling of efficiency, using the ways to enhance self-efficiency including simplifying the behavior, others’ experience on modifying the self-medication, providing ways to control time and stress and its significance in promoting the sense of efficiency and preventing self-medication	Session 4
	Cues to action	Acting according to the advice of the health experts to prevent self-medication, listening to the advice of the husband and relatives to prevent self-medication, listening to the advice of the peers to prevent self-medication. In this session, it was asked the people attending in the session with the experience of self-medication to express their experience and consequences of self-medication.	

After the educational intervention, the phone numbers of the participants or their relatives were recorded to being followed up. They were followed up for three months via phone calls, and eventually, after three months, the questionnaires were distributed and the data for both groups were collected again. The Independent T-Test was used to compare the research units on the basis of demographic data of both intervention and control groups. Furthermore, regarding the inference data, statistical T-Test, Paired T-Test, or their non-parametric equivalents like Mann-Whitney Test and Wilcoxon were used to compare the control and intervention groups before and after intervention. 

## Results

In this study, 200 individuals were examined in two groups of intervention (100 people), and control (100 people). The mean age and the standard deviation of the age of the participants of the present study in the control and intervention groups were 27.45±12.36 and 26.51±11.46 respectively. Using Independent T-Test showed there is no significant difference between the control and intervention groups in terms of age, marital status, education, career, health insurance, and financial status (Table 2).

**Table 2:** Demographic features of women in the Control and Intervention groups

Variable	Intervention group n (%)	Control group n (%)	***p*-value**
Age			0.2
20-24	30 (30)	32 (32)	
25-39	37 (37)	35 (35)	
30-34	23 (23)	20 (20)	
Above35	10 (10)	13 (13)	
Marital Status			0.3
Married	71 (71)	70 (70)	
Unmarried	11 (11)	14 (14)	
Widowed	18 (18)	16 (16)	
Education			0.4
Illiterate elementary	22 (22)	19 (19)	
Guidance	40 (40)	45 (45)	
Diploma and above	38 (38)	36 (36)	
Career			0.2
Housewife	74 (74)	70 (70)	
Working	26 (26)	30 (30)	
Insurance			0.1
Yes	81 (81)	83 (83)	
No	19 (19)	17 (17)	
Financial Status			0.08
Weak	22 (22)	28 (28)	
Average	54 (54)	52 (52)	
Good	24 (24)	20 (20)	

The findings of Wilcoxon test show that the mean scores of knowledge, perceived sensitivity, perceived severity, perceived self-efficiency, perceived benefits, perceived barriers, cues to action and action are statistically meaningful in the intervention group after the intervention (*p*<0.05). As the mean scores of these variables has been increased while the results of this test haven’t shown any significant differences in the control group before and after the intervention (*p*<0.05). The results of the Mann-Whitney proved the lack of any significant difference between control and intervention groups before the intervention, while the difference was meaningful (Table 3). 

**Table 3:** Comparing mean score and Standard Deviation of the variables being studied in Control and Intervention groups

Structure Model	Group	Pre-intervention	Post-intervention	***p*_value** ^*^
		M±SD	M±SD	
Knowledge	Intervention	15.82±3.70	20.94±2.60	*p*<0.01
	Control	14.53±3.24	15.34±3.41	0.03
	*p*_value^**^	0.57	*p*<0.01	
Perceived susceptibility	Intervention	8.65±3.39	13.95±3.11	*p*<0.01
	Control	8.52±3.52	9.02±3.58	0.09
	*p*_value^**^	0.83	*p*<0.001	
Perceived severity	Intervention	9.52±3.31	14.2±2.77	*p*<0.001
	Control	9.48±2.82	10.04±3.22	0.08
	*p*_value^**^	0.37	P<0.001	
Perceived self-efficacy	Intervention	15.48±3.48	22.18±3.53	*p*<0.001
	Control	15.1±3.51	15.84±3.51	0.2
	*p*_value^**^	0.16	*p*<0.001	
				
Perceived benefits	Intervention	12.29±3.35	17.56±2.83	*p*<0.001
	Control	12.40±3.24	13.04±3.79	0.06
	*p*_value^**^	0.12	*p*<0.001	
Perceived barriers	Intervention	11.39±3.47	8.66±3.73	*p*<0.001
	Control	11.54±3.35	10.89±3.87	0.14
	*p*_value^**^	0.18	*p*<0.001	
Cues to action	Intervention	14.49±3.57	19.16±3.23	*p*<0.001
	Control	15.44±3.55	16.09±3.47	0.05
	*p*_value^**^	0.09	*p*<0.001	
Action on self-medication	Intervention	18.52±3.99	14.71±2.31	*p*<0.001
	Control	15.76±3.4	14.34±2.9	0.01
	*p*_value**	0.3	*p*<0.001	

(*) Wilcoxon test, (**) Mann-Whitney

In the present study, regarding the variable of “External cues to action”, for both groups of control and intervention, social media and the physician had the biggest role in receiving the self-medication of women. On the other hand, “interior cues to action” which encourages the individual to take medicine properly, and non-belief into self-medication (47%), had the highest role in both groups of control and intervention (Table 4).

**Table 4:** The frequency distribution of internal and external cues to action

Exterior cues to action	Intervention		Control	
	*n*	%	n	%
RV and radio	24	24	31	31
Book and pamphlet	45	45	40	40
Physician	60	60	65	65
Family and relatives	49	49	45	45
Other mothers referring to health centers	13	13	9	9
Social media	65	65	71	71
Interior cues to action				
Fear of the consequences of self- medication	37	37	32	32
Disbelief in self-medication	47	47	46	46
Favorable general condition	31	31	28	28
Feeling more healthy in self-medication	42	42	35	35

The findings of the women’s performance on taking different medicines in the control and medication groups, before and after the intervention have been shown in percentage. The findings revealed that most drugs women taken through self-medication before the treatment were pain-relievers, cold tablets, and antibiotics, which have been reduced significantly in the self-medication of intervention group after the educational intervention (Table 5).

**Table 5:** the frequency distribution of the self-medication in Intervention and Control groups

Type of the medication	Intervention group		Control group	
	Before	After	Before	After
	***n* (%)**	*n (%)*	***n* (%)**	***n* (%)**
Pain relievers	61 (61)	27 (27)	62 (62)	60 (60)
Cold tablets	53 (53)	27 (27)	55 (55)	50 (50)
Antibiotics	49 (49)	22 (22)	48 (48)	39 (39)
Folic Acid	36 (36)	16 (16)	37 (37)	34 (34)
Acetaminophen	37 (37)	31 (31)	38 (38)	34 (34)
Iron tablet	29 (29)	23 (23)	28 (28)	26 (26)
Multi-vitamins	18 (18)	8 (8)	20 (20)	19 (19)
Herbal medicines	28 (28)	11 (11)	27 (27)	26 (26)
Antihistamine	16 (16)	12 (12)	14 (14)	13 (13)
Antacid	13 (13)	9 (9)	12 (12)	11 (11)
Sleeping pill	10 (10)	8 (8)	11 (11)	9 (9)
Anti-nausea pill	12 (12)	9 (9)	13 (13)	12 (12)
Blood pressure pill	9 (9)	6 (6)	10 (10)	9 (9)
Antipyretic pill	8 (8)	6 (6)	9 (9)	8 (8)

## Discussion

The findings revealed that all components of Health Belief Model had positive meaningful changes after the intervention, being proper indicator of self-medication among women. Furthermore, the social media, physicians and non-belief in self-medication had the biggest role in increasing the knowledge and encouraging to proper use of drugs. Eventually, the findings revealed significant decrease in self-medication of intervention group. In the present study, the knowledge of women has been increased by educating the intervention group. The reason of the difference can be the knowledge of women has been increased by educating the intervention group, which has been in line with what Masoudi Alavi and colleagues,(18) Beijani and colleagues,(19) Xiaosheng Lei.(20) However, there has seen no significant difference in the knowledge scores of the students after the intervention in the study done by Movahed and colleagues.(1) The reason of the difference can be related to the held educational sessions on arbitrary use of the drugs by using different media such as poster, pamphlet, speeches, and slides. Also, the gender and age groups can be the reasons of incompatibility of studies. Therefore, it seems education would be beneficial in modifying people behavior on self-medication. 

The perceived sensitivity of women has been increased after the intervention, which is in line with the studies one by Moghadam,(20) Nikbakht,(21) and Kouhpaye(22) as well. Observing the meaningful significant difference between both groups after the educational intervention in several studies can be prove the importance and effect of the educational intervention on improving the perceived severity of pregnant women in the intervention group, as most mothers, after the intervention, believed that they might had experienced self-medication as well. After the intervention, the mean score of the perceived severity on the consequences of the arbitrary use of drugs has increased significantly in the intervention group. This growing perceived severity has been claimed in other studies like the study done on high school boy students in Manojan,(1) and the study done by Niksadat and colleagues,(21) Beijani and colleagues,(18) as well. In the present study, warning on the serious and sever consequences of the self-medication and drawing people’s attention on loss of health and high treatment expenses have been the two key factors in improving the level of perceived severity of the sample being studied. In the present study, showing the videos of people suffering from consequences of self-medication, and other scenarios prepared to highlight the seriousness of these consequences, and drawing the attention of the participants to the health loss, occurrence of other diseases, and high treatment expenses result in improving the level of the perceived severity of the sample of the participants in this regard.

In the present study, after the educational intervention, the mean score of the perceived benefits has increased for the intervention group, in line with other studies.(18,23) However, it was not in line with the study done by Movahhed and colleagues,(1) Bakhtiar and colleagues,(24) Torshizi and colleagues,(25) The reason of the difference may lie in the fact that there should be adopted proper educational medium in education, regarding the cultural and social differences of the cases being studied. If people noticed the fact that proper use of the medication can reduce the side effects and accelerate the recovery, their perception on the medicine would increase and they would take them properly according to the instructions, while this would never come true but by using the proper educational methods and various media to express the benefits of appropriate manner.

In the present study, the mean score of the perceived barriers has been reduced after the intervention. Moreover, Bakhtiar and colleagues name the perceived barriers as a strong predictor of the self-medication,(25) while Vahedian-Shahroodi and colleagues stated that the perceived barriers can also predict the behaviors relevant to the Calcium intake. In the study done by Shaghaghi and colleagues, the health care costs, lack of adequate time to refer to the doctor, no accessibility of doctors are regarded as the basic barriers of proper use of the medication. Additionally, the present study was similar to the previous ones, necessitates the importance of planning to decrease the barriers.(26) It seems that the perceived barriers are one of the main components of the Health Belief Model, of which the importance has been elaborated in previous studies, and the proper behavior accelerated by its reduction.

In the present study, the social net workings, the doctors and non-belief in the self-medication as the cues for action had the biggest role in improving the knowledge and encouraging to proper use of the drugs. Also, the study of Patrica(27) was in line with this study, introduced doctors and books as the most important exterior cues to action, but the fear of the consequences of the drugs was regarded as the interior cue to action, which was in contrast with the findings of the present study. However, in the study done by Jalilian and colleagues,(28) previous medication, similar prescription and improved symptoms were the greatest reasons of the self-medication among the participants. In the study of Xiaosheng Lei and colleagues the findings were incompatible with the findings of this study, as the relatives’ and friends’ recommendations, Internet, papers and magazines were regarded as the cues to action.(19) In the study done by Movahhed and colleagues,(1) more than half of the participants get the medication information from the doctors, read the drug labels, and a few of them introduced TV, magazines and friends as the reference of getting information on appropriate use of drugs. Furthermore, it is in contrast with the study done in Pakistan, through which, 48% of the participants proposed family as the main source of information on the medication.(27) In the study of Gharouni and colleagues(29) it is revealed that 60% of the patients didn’t read the medicine brochures at all. It is recommended that the doctors have been implemented as a strong leverage in the educational programs, family and friends should be taken as the appropriate guides and supports as well. Regarding the interior cues to action, it is also highlighted the attention to the perceived threats and the harms caused by the arbitrary usage of the drugs.

The findings of the present study revealed that the self-efficiency has increased after the educational intervention, which is in line with the similar studies.(21,30) Paying the special attention to the self-efficiency in the previous studies indicates its significance in the process of education. In the present study, findings revealed that the women performance in terms of arbitrary use of medication has been decreased. The similar results were also shown in the studies done by Shamsi,(24) and Hosseini (32) and Izadirad(33) by a reduction in self-medication. The results of the present study showed that the most drugs arbitrarily used by women before the educational self-medication were pain-relievers, antibiotics and cold tablets. However, in the studied of Jalilian and colleagues,(30) the painkillers, antibiotics, anti-cough and adult tablets were the mostly-used drugs in self-medication. In the study of Bakhtiar,(24) the diseases of headache (77.3%), pain relievers (76.5%), and the cold (62.1%) had the highest amount of the self-medication. The findings of the present study showed that amount of using the antibiotics and cold tablets are high in the self-medication, thus it is recommended to have monitoring the drugstores to prevent from selling the drugs not prescribed and to educate their proper usage according to the prescription. Additionally, the satisfactory and effective results would be achieved by paying attention to the reasons of the self-medication and taking them in the educational programs in terms of arbitrary drug usage.

## Conclusion

The conclusion of this study is that educating on the basis of the Health Belief Model was effective in improving the performance of women referring to the health centers in terms of prevention from the arbitrary use of the drugs. Therefore, it is recommended to have educational intervention by adopting the models of health education, especially, Health Belief Model in preventing and reducing the arbitrary use of the drugs, leading to the improvement in the health behavior of self-medication. Considering the positive effect of training based on the HBM model in preventing self-treatment, the special role of nurses in training and promoting self-treatment literacy based on the model seems necessary. Using this method, nurses can be effective in reducing adverse outcomes and improving women's health. It is recommended to use this educational model as a part of nurses' activities to reduce the problems of hospitalized and treated women.

Strengths and limitations. Implementing the educational model and the type of the study can be regarded as the strengths of the present study. However, the study had limitations including the self-report instrument and limited place, so it ought to be conducted in other settings and places. Additionally, it is recommended to do the research study having the interviews as the data collection instrument. Lack of facilities and supplementary materials, as well as the coordination procedures were regarded as the complexities of the study.
